# Maintenance lenalidomide in newly diagnosed transplant eligible and non-eligible myeloma patients; profiling second primary malignancies in 4358 patients treated in the Myeloma XI Trial

**DOI:** 10.1016/j.eclinm.2023.102099

**Published:** 2023-07-27

**Authors:** John R. Jones, David A. Cairns, Tom Menzies, Charlotte Pawlyn, Faith E. Davies, Rachel Sigsworth, Annamaria Brioli, Matthew W. Jenner, Martin F. Kaiser, Catherine Olivier, Molly Reed, Mark T. Drayson, Roger G. Owen, Kevin D. Boyd, Gordon Cook, Gareth J. Morgan, Graham H. Jackson, NCRI Haemato-Oncology CSG

**Affiliations:** a Brighton and Sussex Medical School, Brighton, UK; b Kings College Hospital, London, UK; c East Sussex NHS Trust, UK; d Cancer Research UK Clinical Trials Unit, Leeds Institute of Clinical Trials Research, University of Leeds, Leeds, UK; e The Institute of Cancer Research, London, UK; f The Royal Marsden Hospital, London, UK; g Perlmutter Cancer Center, NY Langone Health, New York, USA; h Clinic of Internal Medicine C, Greifswald University Medicine, Greifswald, Germany; i University Hospital Southampton NHS Foundation Trust, Southampton, UK; j Clinical Immunology, University of Birmingham, Birmingham, UK; k St James’s University Hospital, Leeds, UK; l Department of Haematology, Newcastle University, Newcastle, UK

**Keywords:** Myeloma, Lenalidomide, Lenalidomide maintenance, Second primary malignancy, SPM, Transplant eligible, Transplant non-eligible

## Abstract

**Background:**

Early trials of long-term lenalidomide use reported an increased incidence of second primary malignancy (SPM), including acute myeloid leukaemia and myelodysplastic syndrome. Later, meta-analysis suggested the link to be secondary to lenalidomide in combination with melphalan.

**Methods:**

Myeloma XI is a large, phase III randomised trial in-which lenalidomide was used at induction and maintenance, in transplant eligible (TE) and non-eligible (TNE) newly diagnosed patients (NCT01554852). Here we present an analysis of SPM incidence and profile the SPM type to determine the impact of autologous stem cell transplantation (ASCT) and lenalidomide exposure in 4358 patients treated on study. Data collection took place from the start of the trial in May 2010, to May 2019, as per the protocol timeline. The Median follow-up following maintenance randomisation was 54.5 and 46.1 months for TE and TNE patients, respectively.

**Findings:**

In the TE pathway, the overall SPM incidence was 7.7% in lenalidomide maintenance patients compared to 3.2% in those being observed (p = 0.006). Although the TNE lenalidomide maintenance patients had the greatest SPM incidence (15.4%), this was not statistically significant when compared to the observed patients (10%, p = 0.10).

The SPM incidence was higher in patients who received lenalidomide at induction and maintenance (double exposure), when compared to those treated with lenalidomide at one time point (single exposure). Again, this was most marked in TNE patients where the overall SPM incidence was 16.9% in double exposed patients, compared to 11.7% in single exposed patients, and 11.2% in patients who did not receive lenalidomide (p = 0.04). This is likely an effect of treatment duration, with the median number of cycles being 27 in the TNE double exposed patients, vs 6 in the single exposure patients.

Haematological SPMs were uncommon, diagnosed in 50 patients (incidence 1.1%). The majority of cases were diagnosed in TE patients treated with lenalidomide maintenance (n = 25, incidence 2.8%), suggesting a possible link with melphalan. Non-melanoma skin cancer incidence was highest in patients receiving lenalidomide maintenance, particularly in TNE patients, where squamous cell carcinoma and basal cell carcinoma were diagnosed in 5.5% and 2.6% of patients, respectively. The incidence of most solid tumour types was higher in lenalidomide maintenance patients.

Mortality due to progressive myeloma was reduced in patients receiving lenalidomide maintenance, noted to be 16.6% compared 22.6% in those observed in TE patients and 32.7% compared to 41.5% in TNE patients. SPM related mortality was low, 1.8% and 6.1% in TE and TNE lenalidomide maintenance patients, respectively, compared to 0.4% and 2.8% in those being observed.

**Interpretation:**

This provides reassurance that long-term lenalidomide treatment is safe and associated with improved outcomes in TE and TNE populations, although monitoring for SPM development should be incorporated into clinic review processes.

**Funding:**

Primary financial support was from 10.13039/501100000289Cancer Research UK [C1298/A10410].

**Research in context**

**Evidence before this study**

Initial studies assessing lenalidomide as both a continuous and maintenance strategy reported an increased risk of second primary malignancy (SPM), including myelodysplastic syndrome (MDS) and acute myeloid leukaemia (AML). Following this, in 2014, a meta-analysis inclusive of seven phase 3 studies and 3218 patients found that the risk of haematological SPM (hSPM) appeared to be linked to lenalidomide when used in combination with oral melphalan. Recently, in 2022, further meta-analysis of SPM incidence in patients receiving lenalidomide treatment for all haematological cancer types, suggests that the SPM risk is unique to patients with myeloma.

**Added value of this study**

Long-term data on SPM incidence in large series of uniformly treated patients is lacking. In addition, no analysis has reported on the profile of SPM developed, or whether lenalidomide treatment at both induction and maintenance poses an additional risk. Lenalidomide is undoubtably effective in myeloma, but a greater understanding of the true SPM risk may further influence its application. Here, we address these unanswered questions, reporting SPM profiles and incidence in 4358 patients treated in the NCRI Myeloma XI trial.

**Implications of all the available evidence**

Lenalidomide is safe and effective as post ASCT maintenance and as continuous therapy in the TNE setting. SPM risk is increased, but myeloma related death is reduced, and SPM mortality is low, providing reassurance for its use. There is no current recommendation regarding the duration of lenalidomide therapy in long-term responders, but this may be taken into consideration for future trial design. We recommend that clinic review should include discussion around SPM, including skin changes and symptoms/signs of solid tumours, particularly in TNE patients, who have an innately greater risk of secondary carcinogenesis.

## Introduction

Outcomes in myeloma continue to improve.^1^, ^2^, ^3^ Autologous stem cell transplantation and novel therapies have been instrumental, but despite this, relapse is almost universal. Attempts to prolong survival have included the use of maintenance. We have previously reported on the use of lenalidomide maintenance in the context of Myeloma XI, the largest trial to date assessing its use in newly diagnosed TE and TNE patients. The trial has shown a progression-free survival benefit in all patient groups and overall survival improvement in patients post-ASCT, who received lenalidomide maintenance.^4^

Early studies assessing lenalidomide maintenance reported an increased incidence of SPM, including MDS and AML.^5^, ^6^, ^7^ This was noted in patients who had undergone ASCT and in those who had not. Subsequent meta-analysis suggested that the use of melphalan in combination with lenalidomide was the main risk factor.^8^ More recently, meta-analysis of 14,058 haematology patients treated with lenalidomide suggests that significant SPM risk is only apparent in the myeloma setting.^9^ Conclusive data is still lacking from large, uniformly treated series of patients, including whether lenalidomide impacts on the profile of SPM developed, and if so, whether the time-point of use or number of cycles received are additional factors.

Interim safety data from the Myeloma XI Trial, inclusive of 2732 patients, with a median follow-up of 34.3 months from trial entry and 24.2 months from maintenance randomisation did not reveal an increased incidence of haematological hSPM in the maintenance lenalidomide patients, irrespective of ASCT status. There was an increased incidence of all cancers, but this was only statistically significant in older, TNE patients, defined as ≥74 years of age. The SPM mortality was 1%, suggesting that the survival benefit associated with lenalidomide use may outweigh the risks.^10^

Here we report the long-term incidence of SPM in Myeloma XI, inclusive of all 4358 treated patients. We determine SPM incidence and profile the types of malignancies seen in TE and TNE patients, including whether there is an impact on SPM development in patients treated with lenalidomide at both induction and maintenance. This forms the largest assessment of SPM profiling in a uniformly treated, newly diagnosed TE and TNE patients.

## Methods

### Study design and participants

Myeloma XI is a phase III, multicentre, randomised, open-label and adaptive design trial conducted in 110 National Heath Centre Hospitals (NCT01554852). As per the statistical analysis plan, the SPM analysis includes the safety population, not the intention to treat. This analysis reports SPM incidence in all patients who received at least one cycle of trial prescribed treatment.

All second cancers developed during the trial and in patients who had left the trial were included in the analysis. Second cancer data was collected prospectively from the start of the Trial, including a review of histological reports and/or imaging for all cases, to ensure all confirmed cases were included. All cancer types were counted, including non-melanoma skin cancers (NMSC), solid tumours and haematological malignancies.

All patients recruited were assessed for transplant eligibility and then randomised between thalidomide, lenalidomide or carfilzomib (TE only) containing induction regimes. Patients not achieving at least a very good partial remission, may have also received bortezomib intensification prior to ASCT or maintenance randomisation, as per protocol, Supplementary Figure S1. Patients were eligible for maintenance randomisation if they achieved a minimal response or better (minimal response defined as between 25 and 49% reduction in presentation paraprotein or involved light chain). In patients post ASCT, maintenance randomisation was conducted 100 days after cell return. TNE patients entered maintenance randomisation following induction treatment, ±bortezomib intensification. Randomisation was between lenalidomide (±vorinostat) and observation only, until progression. Those receiving lenalidomide maintenance were monitored monthly, whilst patients being observed were monitored every two months until 2 years and then every 3 months thereafter.

### Statistical analysis

All statistical analyses were undertaken in SAS (version 9.4; SAS Institute, Cary, NC) according to the Trial Statistical Analysis Plan. Cumulative incidence function curves were estimated by non-parametric maximum likelihood estimation^11^ and plotted overall, by pathway and by treatment arm. The Pepe–Mori test^12^ for equality of cumulative incidence functions was used to compare time to first SPM by treatment allocation, and other variables, with unrelated deaths as a competing risk. Person-years on trial was calculated as the sum over all patients receiving at least one dose of study treatment of the time in years from randomisation to death or last date known to be alive. Incidence rates were calculated with the number of events as the numerator and the number of person-years on trial as the denominator. Confidence intervals for incidence rate were calculated using approximations for the Poisson distribution. All statistical tests were two-sided and called significant at the 5% level.

### Ethical statement

All patients included provided written informed consent. The study is now closed for accrual, but follow-up continues for long-term analysis. The study was approved by the national ethics review board (National Research Ethics Service, London, UK), institutional review boards of the participating centres, and the competent regulatory authority (Medicines and Healthcare Products Regulatory Agency, London, UK), and was undertaken according to the Declaration of Helsinki and the principles of Good Clinical Practice as espoused in the Medicines for Human Use (Clinical Trials) Regulations.

### Role of the funding source

JJ, DC and TM had access to the data set. All study authors agreed on the decision to submit this manuscript for publication. The funders of the study have not been involved in data collection, data analysis, data interpretation or writing of the paper.

## Results

### Study characteristics

Since May 2010, 4358 patients entered and received induction therapy. Median follow up at the data cut-off was 60 months (interquartile range 47–76). Of the 4358 patients that initiated treatment, 2532 entered the TE pathway and 1826 entered the TNE pathway. Within the TE pathway 1008 patients received CTD, 1014 received CRD and 510 received KCRD induction. In the TNE pathway 910 patients received CTDa and 916 received CRDa, Supplementary Table S1. A total of 2274 patients were randomised to maintenance, with 1368 receiving lenalidomide (±vorinostat) and 906 were observed (median 24 cycles (range 1–97)). Each cycle lasted four weeks, with lenalidomide given for three weeks and one week with no treatment.

The median follow-up since maintenance randomisation is 46 and 55 months, for TE and TNE patients, respectively. A breakdown of follow-up times according to treatments received in the TE and TNE pathway is detailed in Supplementary Table S2. The median age of patients entering the TE and TNE pathways was 61 years (range 28–75) and 74 years (range 54–92), respectively. Data cut-off for this analysis was 31/05/2019, as per the protocol timeline.

### SPM summary

Three hundred and seventy-six SPM in 318 patients were identified. Of the 318 patients, 277 had 1 SPM and 41 patients had 2 or more. The median age at SPM diagnosis was 73 years (range 50–90) and the median time to first diagnosis from induction randomisation was 35.7 months (range 1.4–96.6).

Overall trial-related SPM incidence was 7.3%, with an incidence rate of 2.4 per 100 person years. The cumulative incidence of SPM in the whole trial population was 4.1% (95% confidence interval (CI) 3.5%–4.7%), 7.4% (95% CI 6.6%–8.3%) and 10.9% (95% CI 9.6–12.2%) at 3, 5 and 7 years, respectively.

One hundred and thirty-eight TE (incidence 5.5%) patients developed an SPM compared to 180 TNE patients (incidence 9.9%), Consort Diagrams A and B. Incidence rate per 100 patient-years for the TE and TNE pathways was 1.6 and 3.7, respectively, Table 1. The median time to first SPM diagnosis from induction randomisation was 40 months (range 2–85), and 32 (range 1–97) for the TE and TNE pathways. The median age at first SPM diagnosis was 68 years (range 50–76) for the TE patients and 77 years (range 65–90) for TNE patients.

**Consort Diagram A:**
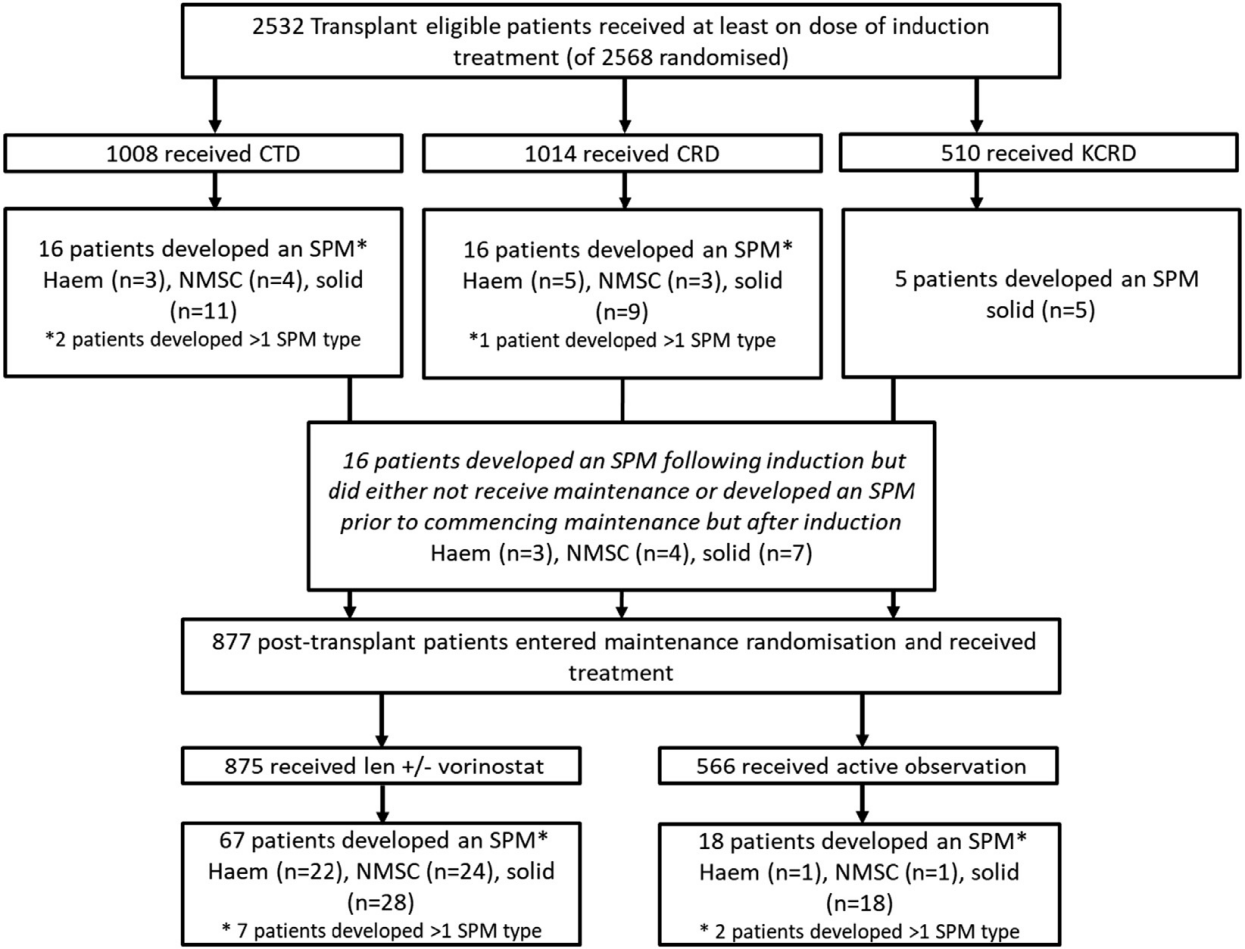
**Transplant eligible consort diagram detailing the number of patients randomised and number of SPMconfirmed.**

**Consort Diagram B:**
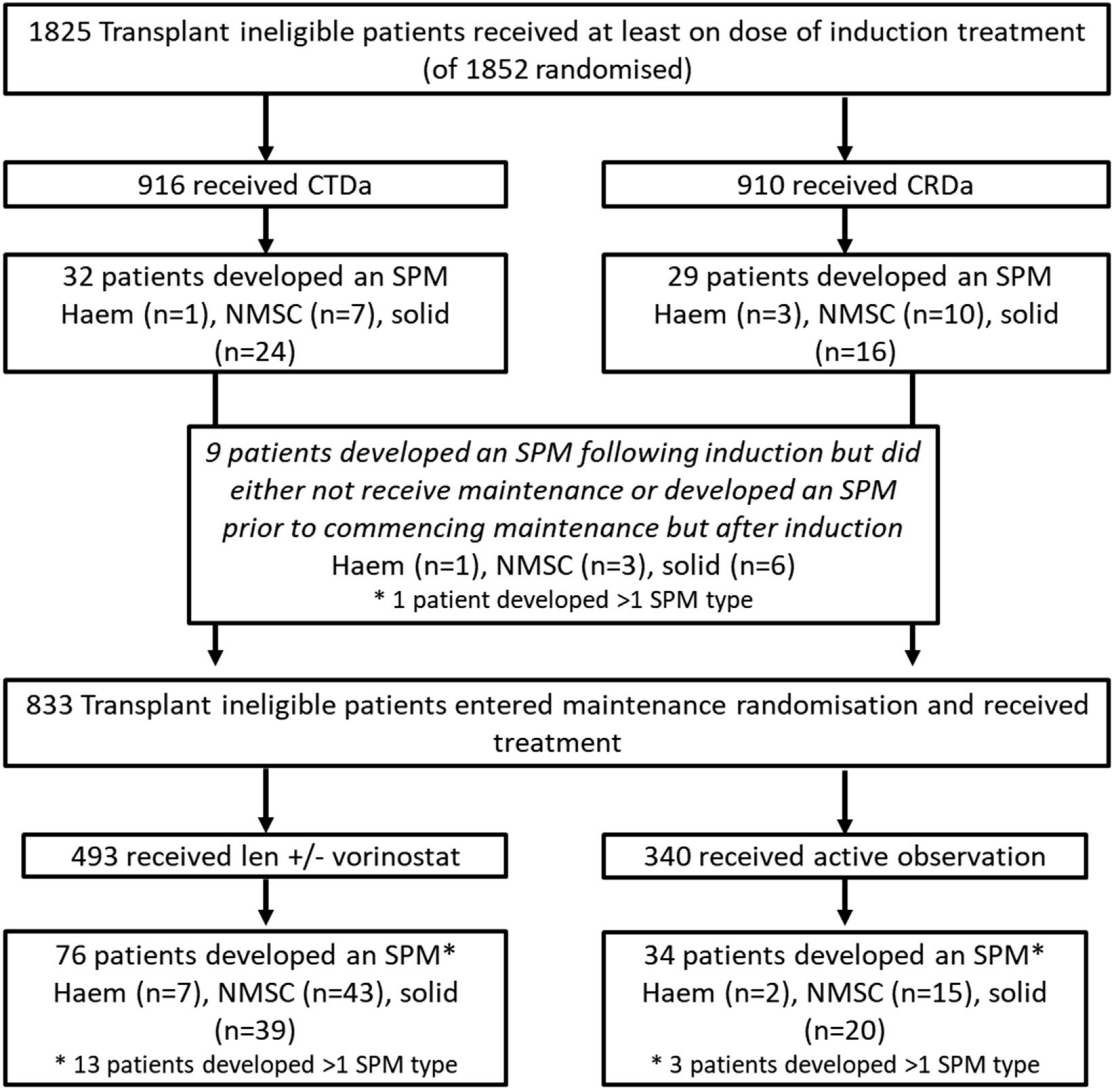
**Transplant non-eligible consort diagram detailing the number of patients randomised and number of SPM confirmed.** Consort diagram outlining the number of patients randomized according to treatment allocation in the transplant eligible patients (consort A) and non-eligible patients (consort B). The number and type of SPM (haematological, NMSC and solid) developed during each treatment phase and according to treatment received is detailed. Abbreviations: CTD, cyclophosphamide, thalidomide and dexamethasone; CRD, lenalidomide, cyclophosphamide and dexamethasone; KCRD, carfilzomib, cyclophosphamide, lenalidomide and dexamethasone; len, lenalidomide; vori, vorinostat; SPM, second primary malignancy; haem, haematological; NMSC, non-melanoma skin cancer; solid, solid tumours.

**Table 1:** SPM incidence according to pathway and treatment received.

	Whole trial	p^a^	TE	p^a^	TNE	p^a^
**Overall incidence (IR) and 7-year cumulative incidence (CI) according to induction (%)**						
- Whole trial	Overall IR 7.3 7-year CI 10.9 (n = 4358; 318 SPM)	–	Overall IR 5.5 7-year CI 9.0 (n = 2532; 138 SPM)	–	Overall IR 9.9 7-year CI 13.4 (n = 1825; 180 SPM)	–
- Lenalidomide	Overall IR 8.0 7-year CI 11.5 (n = 1930; 154 SPM)	0.39	Overall IR 6.2 7-year CI 9.3 (n = 1014; 63 SPM	0.08	Overall IR 9.9 7-year CI 14.3 (n = 916; 91 SPM)	0.47
- Thalidomide	Overall IR 7.56 7-year CI 10.3 (n = 1918, 145 SPM)		Overall IR 5.56 7-year CI 8.3 (n = 1008, 56 SPM)		Overall IR 9.78 7-year CI 12.5 (n = 910; 89 SPM)	–
- Carfilzomib and lenalidomide^b^	–	–	Overall IR 3.7 5-year CI 8.1% (n = 510, 19 SPM)	–	–	–
**Overall incidence rate (IR) and 5-year cumulative incidence (CI) according to maintenance (%)**						
- Lenalidomide ± vorinostat (%)	Overall IR 10.5 5-year CI 13.1 (n = 1368; 143 SPM)	0.013	Overall IR 7.7 5-year CI 10.5 (n = 875; 67 SPM)	0.006	Overall IR 15.4 5-year CI 17.2 (n = 493; 76 SPM)	0.10
- Observation only (%)	Overall IR 5.7 5-year CI 6.6 (n = 906; 52 SPM)		Overall IR 3.2 5-year CI 4.1 (n = 566; 18 SPM)		Overall IR 10 5-year CI 10.2 (n = 340; 34 SPM)	
- TNE ≤74 years lenalidomide ± vorinostat (%)	–	–	–	–	Overall IR 9.2 5-year CI 16.6 (n = 262; 24 SPM^c^)	0.30
- TNE ≤74 years observation only (%)	–	–	–	–	Overall IR 6.9 5-year CI 10.8 (n = 175; 12 SPM)	
- TNE >74 years lenalidomide ± vorinostat (%)	–	–	–	–	Overall IR 22.5 5-year CI 17.7 (n = 231; 52 SPM^c^)	0.09
- TNE >74 years observation only (%)	–	–	–	–	Overall IR 13.9 5-year CI 9.5 (n = 165; 23 SPM)	
**SPM incidence per 100 person-years after induction randomisation (confidence interval)**						
Overall	2.4 (2.1, 2.6)	–	1.6 (1.4, 1.9)	–	3.7 (3.3, 4.3)	
CTD	–	–	1.6 (1.2, 2.0)	–	–	–
CRD	–	–	1.8 (1.5, 2.3)	–	–	–
KCRD	–	–	1.2 (0.8, 1.8)	–	–	–
CTDa	–	–	–	–	3.5 (2.9, 4.3)	–
CRDa	–	–	–	–	3.9 (3.2, 4.7)	–
**SPM incidence per 100 person-years after maintenance randomization (confidence interval)**						
Overall	3.0 (2.6, 3.4)	–	2.0 (1.6, 2.4)	–	4.8 (4.1, 5.7)	–
Active observation	–	–	1.0 (0.6, 1.5)	–	3.3 (2.4, 4.5)	–
Lenalidomide	–	–	2.2 (1.7, 2.9)	–	6.8 (5.5, 8.4)	–
Lenalidomide + vorinostat	–	–	3.7 (2.5, 5.3)	–	3.1 (1.8, 5.4)	–

Data relates to the number of patients who developed an SPM, not the total number of SPMs reported.

Abbreviations: CTD, cyclophosphamide, thalidomide and dexamethasone; CRD, lenalidomide, cyclophosphamide and dexamethasone; KCRD, carfilzomib, cyclophosphamide, lenalidomide and dexamethasone; len, lenalidomide; vori, vorinostat.

a Pepe–Mori significance for 7 and 5 year cumulative incidence data.

b 5 year incidence reported for KCRd due to shorter follow-up. KCRd was added as a randomization option for TE patients later in the trial.

c One patient developed as SPM aged 74 and also aged 75 so was included in both the ≤74 and >74 groups.

### Timing of SPM development

#### Induction

**Fig. 1:**
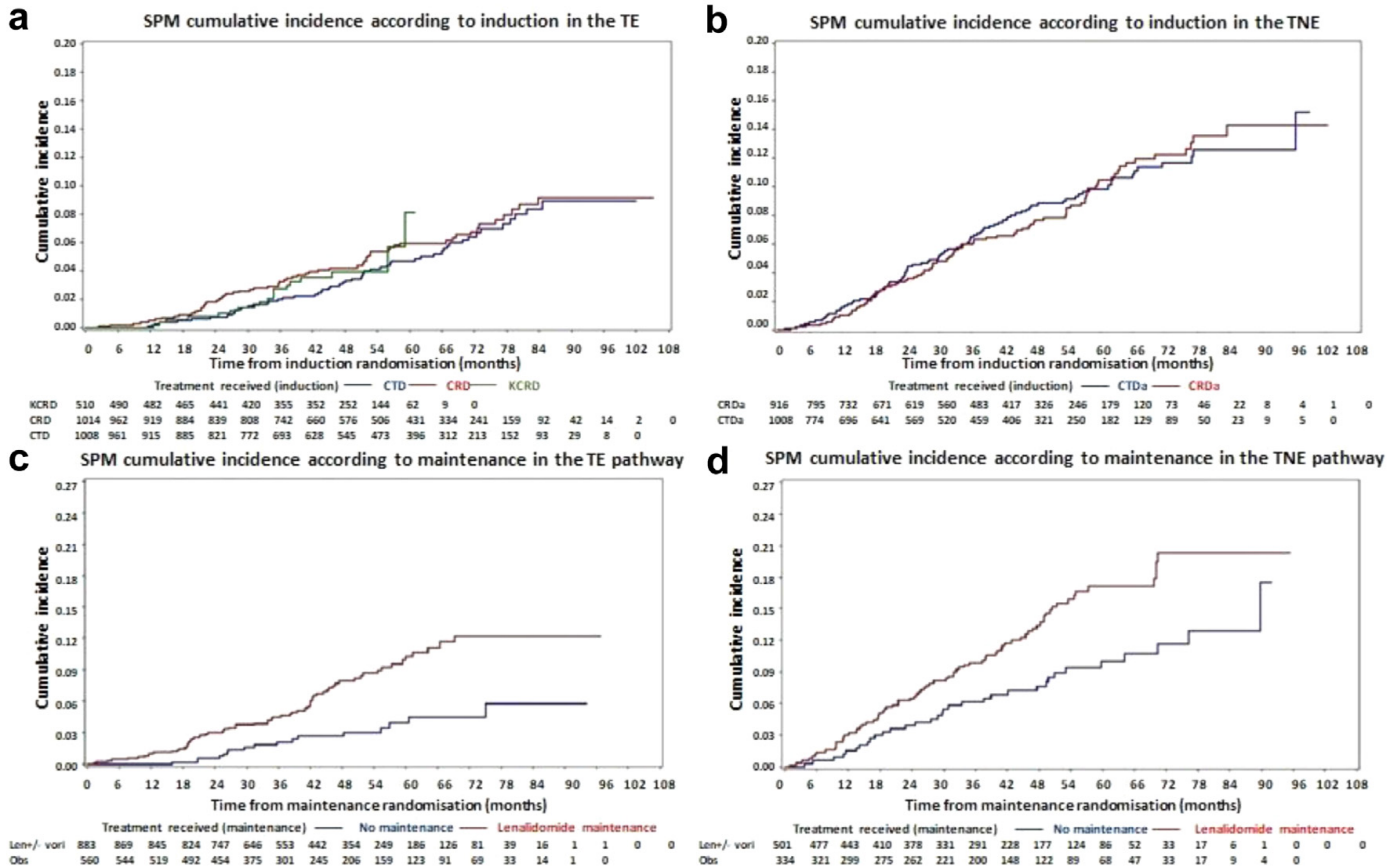
**Cumulative incidence of SPM according to induction treatment received.** a) The cumulative incidence of SPM in the TE pathway according to induction. The SPM incidence in patients receiving CTD was 2.0%, 4.7% and 8.3% at 3, 5 and 7 years. In patients receiving CRD the incidence was 3.1%, 5.8% and 9.3% at 3, 5 and 7 years (Pepe–Mori p = 0.08). The data relating to KCRD induction is less mature but the incidence at 3 and 5 years was 2.7% and 8.1% years. b) The cumulative incidence of SPM in the TNE pathway according to induction. The SPM incidence in patients receiving CTDa was 6.5%, 9.8% and 12.5% at 3, 5 and 7 years, respectively. The incidence in patients receiving CRDa was 6.0%, 10.4% and 14.3% at 3, 5 and 7 years, respectively (Pepe–Mori p = 0.47). c) The cumulative incidence of SPM in the TE pathway according to maintenance. The SPM incidence in patients being observed was 2.1%, 4.1% and 5.8% at 3, 5 and 7 years. In patients randomised to lenalidomide ± vorinostat the incidence was 4.5%, 10.5% and 12.2% (Pepe–Mori p = 0.006). d) The cumulative incidence of SPM in the TNE pathway according to maintenance. The SPM incidence in patients being observed was 6.2% and 10.2% and at 3 and 5 years. In patients randomised to lenalidomide ± vorinostat the SPM incidence was 9.9% and 17.2% at 3 and 5 years (Pepe–Mori p = 0.10).

Within the TE pathway 37 patients (incidence 1.5%) developed an SPM during induction. The cumulative incidence in patients who received CTD was 2.0%, 4.7%, and 8.3% at 3, 5 and 7 years, respectively. This was comparable to the incidence in patients who received CRD, being 3.1%, 5.8% and 9.3% (Pepe–Mori p = 0.08). Patients receiving KCRD had an SPM incidence of 2.7% and 8.1% at 3 and 5 years, Fig. 1a.

Within the TNE pathway 61 patients (incidence 3.3%) developed an SPM during induction. Those treated with CTDa had a cumulative SPM incidence of 6.5%, 9.8% and 12.5% at 3, 5 and 7 years, respectively. Patients treated with CRDa had a cumulative SPM incidence of 6.0%, 10.4% and 14.3% at the same time points (Pepe–Mori p = 0.47), Fig. 1b.

This data shows that SPM incidence does not appear to be significantly affected by the treatment given at induction, although SPM incidence is higher in older patients.

#### Lenalidomide maintenance vs observation

Eighty-five (incidence 5.9%) TE patients developed an SPM during maintenance. Patients who received lenalidomide ± vorinostat (n = 875) maintenance had a cumulative SPM incidence of 4.3%, 10.5% and 12.4% at 3, 5 and 7 years. In observed patients (n = 566) the cumulative SPM incidence was 2.2%, 4.1% and 5.9% at 3, 5 and 7 years (Pepe-Mori p = 0.006) Fig. 1c. The incidence rate per 100 patient years was 2.2 (95% CI 1.7–2.9), 3.7 (95% CI 2.5–5.3) and 1.0 (95% CI 0.7–1.6) for those receiving lenalidomide, lenalidomide plus vorinostat and observation, respectively, Table 1.

One hundred and ten (incidence 13.2%) TNE patients developed an SPM during maintenance. In those who received lenalidomide ± vorinostat maintenance (n = 493) the cumulative SPM incidence was 9.8% and 17.2% at 3 and 5 years, respectively. In observed patients (n = 340) the cumulative SPM incidence was 6.4% and 10.2% for 3 and 5 years (Pepe-Mori p = 0.10), respectively, Fig. 1d. Few patients in the TNE pathway reached 7 years following maintenance (n = 15). The incidence rate per 100 patient years was 6.8 (95% CI 5.5–8.4), 3.1 (95% CI 1.8–5.4) and 3.3 (95% CI 2.4–4.5) for those receiving lenalidomide, lenalidomide plus vorinostat and observation, respectively, Table 1.

In the TE pathway, the median time to first SPM development in lenalidomide maintenance patients (from maintenance randomisation) was 35 months (range 1–69) and 31 months in those being observed (range 1–75). In the TNE pathway, the median time to first SPM development was 28 months (range 2–71) in those receiving lenalidomide, compared to 29 months (range 4–90) in those being observed.

These data reveal that SPM incidence is higher in patients who received lenalidomide maintenance, compared to those being observed.

#### Lenalidomide maintenance in advanced age

The median age of the TNE patients was 74 years and, therefore, this was used as a cut-off for defining advanced age. The cumulative SPM incidence in TNE patients ≤74 who were randomised to lenalidomide ± vorinostat was 8.7% (95% CI 5.2–12.2) at 3 years and 16.6% (95% CI 11.6–21.7) at 5 years. In observed patients, the cumulative incidence was 7.7% (95% CI 3.7–11.7) and 10.8% (95% CI 5.9–15.8) at 3 and 5 years, respectively (Pepe Mori p = 0.30), Supplementary Figure S2a.

In the TNE patients >74 the cumulative SPM incidence in patients who received lenalidomide ± vorinostat was 11.1% (95% CI 7.0–15.2) and 17.7% (95% CI 12.2–23.2) at 3 and 5 years, respectively. In those randomised to observation the cumulative incidence was 4.9% (95% CI 1.6–8.3) and 9.5% (95% CI 4.5–14.6) at 3 and 5 years, respectively (Pepe Mori p = 0.09), Supplementary Figure S2b.

These data illustrate the impact of age on SPM development, with the greatest incidence noted in patients >74 receiving lenalidomide.

### SPM type

#### Haematological malignancies

Fifty patients (incidence 1.1%) developed a hSPM, 36 within the TE pathway (incidence 1.4%) and 14 (incidence 0.8%) in the TNE pathway. Of the 50 patients, 32 received lenalidomide maintenance, 25 in the TE pathway (incidence 2.8%) and 7 in the TNE pathway (incidence 1.4%). The median time to hSPM development in the TE and TNE patients was 51.8 months (range 12.4–82.9) and 42.3 months (range 22.9–78.9). The median age at time of hSPM diagnosis was 79 (range 72–83) in TNE patients and 68 (range 50–76) in TE patients. The most diagnosed hSPM were MDS (N = 21), AML (n = 13), DLBCL (n = 6) and B-ALL (n = 4) Supplementary Table S3a and S3b.

#### Solid malignancies

One hundred and eighty-three solid malignancies (including melanoma and fibroxanthoma but excluding BCC and SCC) in 177 patients were confirmed (incidence 4.1%). Seventy-six (incidence 3%) TE patients developed an SPM compared to 101 (incidence 5.5%) TNE patients. The median age of SPM diagnosis was 67 (range 53–76) years and 77 (range 65–90) years for the TE and TNE pathways, respectively. The median time to SPM diagnosis from induction randomisation was 38.8 (range 2.4–84.7) months in the TE pathway and 32.0 (range 1.4–97.1) months in the TNE pathway. The most diagnosed were prostate (22 cases), breast (20), colon (19), lung (16) and melanoma (16), Supplementary Table S4a and S4b.

#### Non-melanoma skin cancers

One hundred and forty-three NMSC were diagnosed in 106 patients (incidence 2.4%), Supplementary Table S5a and S5b. Thirty-three (incidence 1.3%) patients were enrolled to the TE pathway and 73 (incidence 4.0%) to the TNE pathway. The median time to diagnosis from entry in the TE patients was 42 (range 12.9–85.8) months with a median age at diagnosis of 69 (range 50–75). The median time to diagnosis from entry in the TNE patient was 37.3 (range 3.7–78.1) months, with a median age of 78 (range 67–89).

In summary, these data illustrate that hSPM were uncommon and mainly diagnosed in TE patients. Solid tumours and NMSC formed the majority of SPM, with the greatest incidence in TNE patients.

#### Impact of lenalidomide maintenance on SPM type

**Fig. 2:**
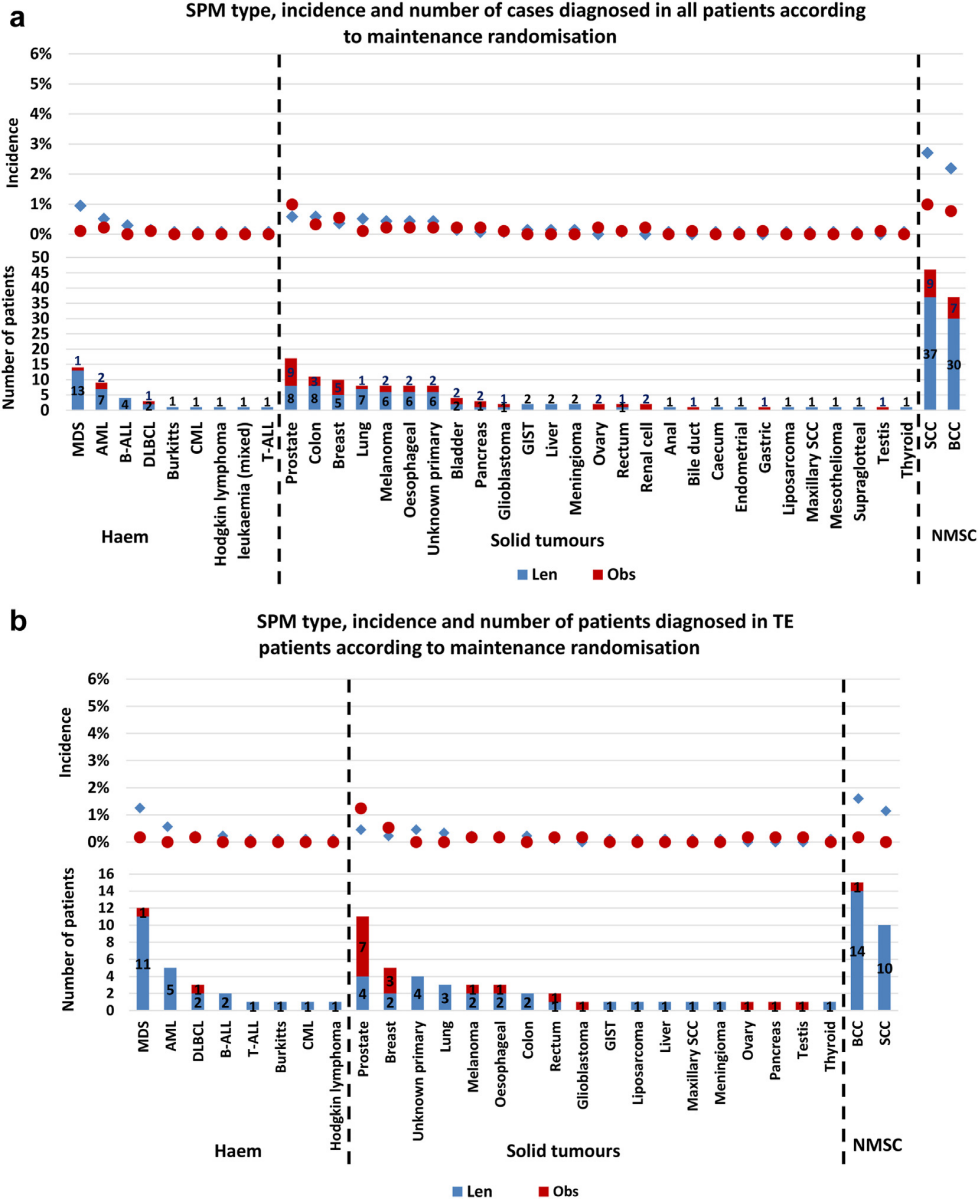

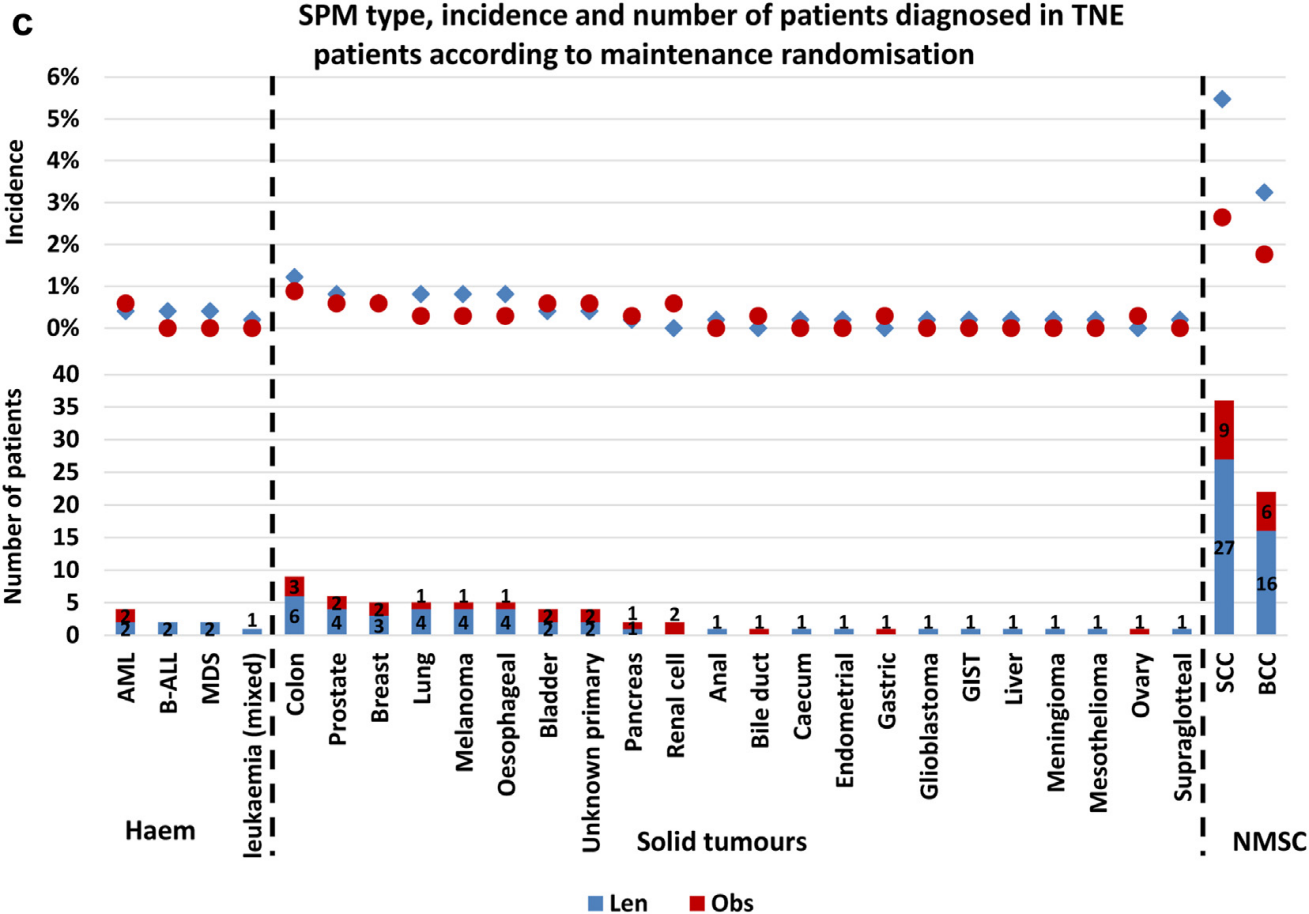
**Number and incidence of SPM type according to maintenance randomisation.** a) The type, incidence and number diagnosed in all trial patients randomised through maintenance. The overall incidence of all cancer types was low. In the lenalidomide maintenance patients, only SCC (2.7%) and BCC (2.2%) were diagnosed in >1% of patients. No cancers were diagnosed in >1% of the patients being observed. Haematological malignancies were almost exclusively seen in the lenalidomide maintenance patients, with MDS and AML forming the majority of cases. Of the 38 cancer types, more cases were noted in the lenalidomide treated patients, except for prostate, pancreas, ovary, renal cell, bile duct, gastric and testes, where there was either 1 or 2 extra cases in those randomised to observation. b) The type, incidence and number diagnosed in TE pathway patients randomised through maintenance. The overall incidence of most SPM types was low, noted in <1% of patients. In the lenalidomide maintenance patients, BCC (1.6%), MDS (1.3%) and SCC (1.1%) were noted in >1%. In the observation series only prostate cancer (1.2%) was diagnosed in >1%. Haematological malignancies were almost confined to the patients receiving lenalidomide, with most cases being AML and MDS. Only two haematological SPM were diagnosed in the observed patients, compared to 24 in those receiving lenalidomide. BCC and SCC were also almost completely confined to lenalidomide maintenance patients. Most other cancer types were diagnosed in a small number of patients, although the majority in those receiving lenalidomide. c) The type, incidence and number diagnosed in TNE pathway patients randomised through maintenance. SCC and BCC were the most diagnosed SPM in both the lenalidomide and observation patients, although most cases were noted in the lenalidomide maintenance patients, where the overall incidence of SCC and BCC was 5.5% and 2.6%, respectively. Only eight patients were diagnosed with a haematological SPM, 7 of which were in the lenalidomide maintenance patients. More SPM and SPM types were diagnosed in the lenalidomide maintenance patients. Abbreviations: MDS, myelodysplastic syndrome; AML, acute myeloid leukaemia; B-ALL, B lymphocyte acute lymphoblastic leukaemia; DLBCL, diffuse large B cell lymphoma; CML, chronic myeloid leukaemia; T-ALL, T lymphocyte acute lymphoblastic leukaemia; GIST, gastrointestinal stromal tumour; SCC, squamous cell carcinoma; BCC, basal cell carcinoma; Haem, haematological malignancy; NMSC, non-melanoma skin cancer; solid, solid tumour.

Thirty-eight different cancer types were diagnosed in patients who had been through maintenance randomisation. Of the 38 different types, 17 were noted in both observation and lenalidomide maintenance groups, 16 were confined to only lenalidomide maintenance patients and five solely in those being observed. The incidence of each cancer type was low. In all lenalidomide maintenance patients, only squamous cell carcinoma (SCC) and basal cell carcinoma (BCC) were diagnosed in >1% of patients. No individual SPM type was diagnosed in >1% of patients being observed, Fig. 2a.

In TE patients, 28 different cancer types were diagnosed. Most cases were noted in the lenalidomide maintenance patients, although the overall incidence of all cancer types was low. Only BCC (1.6%), MDS (1.3%) and SCC (1.1%) were diagnosed in >1% of lenalidomide maintenance patients. In the observed patients only prostate cancer (1.2%) was diagnosed in >1%. Haematological SPMs were almost confined to the lenalidomide maintenance patients, particularly MDS (1.3%) and AML (0.6%), diagnosed in a total of 16 patients, compared to 1 (MDS, 0.2%) patient being observed, Fig. 2b.

In TNE patients, 28 different cancer types were diagnosed. Few hSPMs were diagnosed, with only 8 cases, 7 in patients receiving maintenance lenalidomide. The incidence of NMSC was marked, particularly in the lenalidomide patients, with an SCC incidence of 5.5% and BCC incidence of 2.6%, Fig. 2c.

These data show that the incidence of each SPM type was low, apart from NMSC which were common, particularly in the TNE lenalidomide maintenance patients.

### Impact of treatment with lenalidomide at presentation and maintenance

**Fig. 3:**
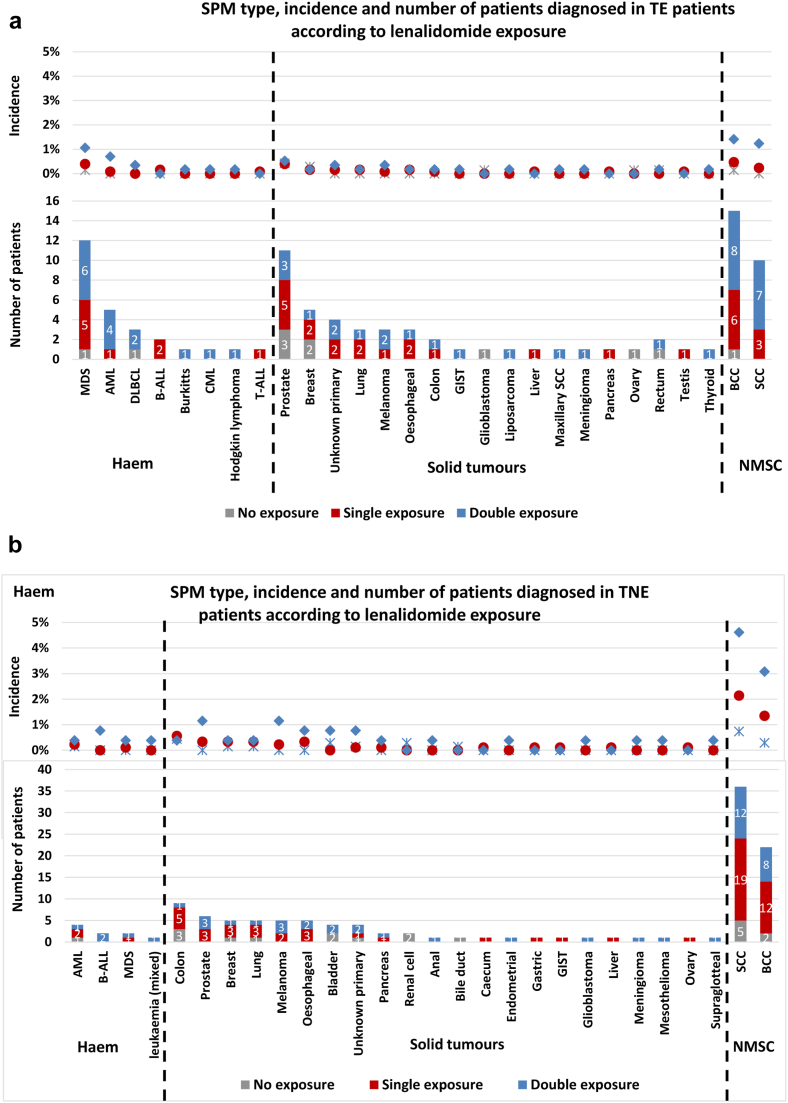
**SPM incidence and profile according to lenalidomide exposure at induction and maintenance.** a) SPM type, incidence and number of patients diagnosed in TE patients following maintenance randomisation according to lenalidomide exposure. Patients not treated with lenalidomide (grey star/bar) during the trial relates to those who received CTD induction and randomised to observation (n = 701). Patients who received lenalidomide at one time-point i.e., single exposure (red circle/bar), received KCRd or CRD at induction and randomised to observation, or patients who received CTD and were randomised to lenalidomide maintenance (total = 1263). The blue diamond/bar details SPM cases diagnosed in patients exposed to lenalidomide at both induction and maintenance (double exposure) and includes patients who received CRD or KCRD and were randomised to lenalidomide maintenance (n = 568). Most SPM were diagnosed in patients who had been exposed to lenalidomide, with the greatest incidence noted in the double exposed group. Haematological SPMs were almost confined to the lenalidomide treated patients, with a greater incidence in those who had received lenalidomide at induction and maintenance. Few SPM were noted in the patients who were not exposed to lenalidomide. b). SPM type, incidence and number of patients diagnosed in TNE patients following maintenance randomisation according to lenalidomide exposure. Patients not treated with lenalidomide (grey star/bar) during the trial includes those who received CTDa induction and were randomised to observation (n = 677). Patients who received lenalidomide at one time-point i.e., single exposure (red circle/bar), received CRDa at induction and were randomised to observation, or patients who received CTDa and were randomised to lenalidomide maintenance (total = 899). The blue diamond/bar details SPM cases diagnosed in patients exposed to lenalidomide at both induction and maintenance (double exposure) and includes patients who received CRDa and lenalidomide maintenance (n = 260). Non-melanoma skin cancers dominated, particularly in the lenalidomide exposed patients, with the greatest incidence in the double exposed group. Haematological SPMs were rare, although almost confined to the lenalidomide treated patients. Solid tumours were also noted more frequently in the lenalidomide treated patients, with the greatest incidence in those who were exposed at induction and maintenance. Abbreviations: MDS, myelodysplastic syndrome; AML, acute myeloid leukaemia; B-ALL, B lymphocyte acute lymphoblastic leukaemia; DLBCL, diffuse large B cell lymphoma; CML, chronic myeloid leukaemia; T-ALL, T lymphocyte acute lymphoblastic leukaemia; GIST, gastrointestinal stromal tumour; SCC, squamous cell carcinoma; BCC, basal cell carcinoma; Haem, haematological malignancy; NMSC, non-melanoma skin cancer; solid, solid tumour; CTD, cyclophosphamide, thalidomide and dexamethasone; CRD, lenalidomide, cyclophosphamide and dexamethasone; KCRD, carfilzomib, cyclophosphamide, lenalidomide and dexamethasone; len, lenalidomide; vori, vorinostat.

Patients were grouped according to no lenalidomide treatment, single exposure, i.e., at induction or maintenance, or double exposure i.e., at both induction and maintenance. In TE patients the SPM incidence was greater in the double exposed patients, although this was not statistically significant (non-exposed overall incidence 4.2% vs single exposed 5.0% (p = 0.51), single exposed vs double exposed 7.6% (p = 0.20)). Despite this, the trial related incidence of individual SPM types was low, being <1%, except for BCC (1.4%), SCC (1.2%) and MDS (1.1%) noted in >1% of patients treated with lenalidomide and both induction and maintenance. Haematological SPMs were almost confined to the lenalidomide-treated patients, with most in the double exposed group, Fig. 3a. The median number of lenalidomide cycles received in the single exposure group was 6 (range 1–96) and 34 (range 4–97) in the double exposure group.

In the TNE patients, there was also a greater SPM incidence in the double exposed patients (non-exposed overall incidence 11.2% vs single exposed 11.7% (p = 0.16), single exposed vs double exposed 16.9% (p = 0.04)). Non-melanoma skin cancers were common, particularly in the double exposed patients, with an SCC incidence of 4.6% and BCC incidence of 3.1%. No SPM type was diagnosed in >1% of non-exposed patients. In the double exposed patients, prostate cancer (1.2%) and melanoma (1.2%) were also noted in >1% of patients, Fig. 3b. The median number of lenalidomide cycles received in the single exposure group was 6 (range 1–92) and 27 (range 6–101) in the double exposure group.

These data show that the incidence of SPM is highest in patients who received lenalidomide at both induction and maintenance. This is likely to be as a consequence of treatment duration.

### Deaths secondary to SPM and other causes

At the median follow-up point of 54.5 months and 46.1 months for TE and TNE patients, respectively, we determined the outcome for all patients, including the proportion of patients who died because of SPM.

In TE patients treated with lenalidomide maintenance, 13 (incidence 1.8%) deaths were secondary to an SPM, compared to 121 (incidence 16.6%) myeloma-related and 18 (incidence 2.5%) non-myeloma related deaths. This compared to 2 (incidence 0.4%) SPM deaths, 117 (incidence 22.6%) myeloma-related and 19 (incidence 3.7%) non-myeloma related deaths in the observed patients.

**Fig. 4:**
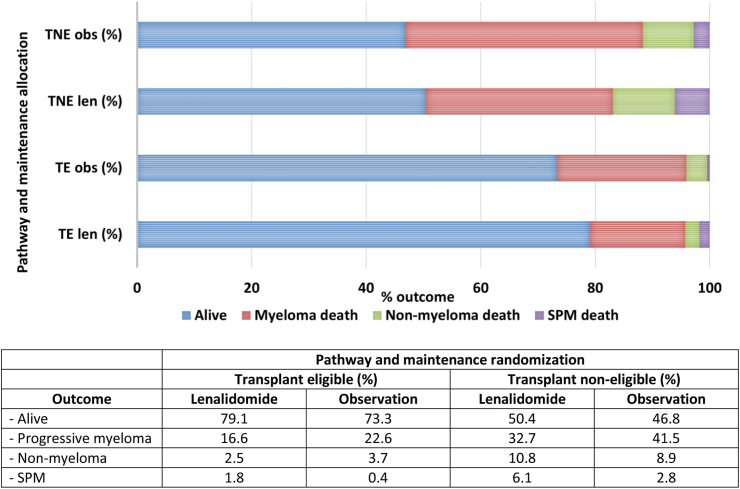
**Outcome of patients treated in the TE and TNE pathway according to maintenance randomisation.** Outcomes according to pathway and maintenance randomisation. In both the TE and TNE pathways, a greater proportion of patients receiving lenalidomide were alive at the data cut-off, compared to those who were being observed. The main cause of death in all groups was progressive myeloma, although the incidence was lower in both the TE and TNE lenalidomide maintenance series, compared to the patients being observed. Non myeloma related death was low in patients treated in the TE pathway, reported in 2.5% of the lenalidomide maintenance patients and 3.7% of patients being observed. The non-myeloma death rate was higher in TNE patients, reported in 10.8% of those receiving lenalidomide maintenance and 8.9% of patients being observed. The SPM death rate was low in all groups, but highest in the TNE patients treated with maintenance lenalidomide, where 6.1% of patients died as a consequence of a second cancer.

In TNE lenalidomide maintenance patients, 25 (incidence 6.1%) died due to a SPM, 133 (incidence 32.7%) due to progressive myeloma and 44 (incidence 10.8%) due to other causes. In the observed TNE patients, 9 (incidence 2.8%) died due to an SPM, 131 (incidence 41.5%) due to progressive myeloma and 28 (incidence 8.9%) secondary to non-myeloma related issues, Fig. 4.

This data shows that lenalidomide maintenance is associated with reduced myeloma related mortality and a low risk of SPM related death.

## Discussion

This is the largest single trial analysis of SPM incidence associated with lenalidomide as both an induction and maintenance treatment option, in newly diagnosed TE and TNE eligible, uniformly treated patients.

Significantly, in TE patients, the overall SPM incidence rate was 7.7% in those randomised to lenalidomide maintenance, compared to 3.2% in those being observed (p = 0.006). Importantly however, there was a low risk of SPM related death, noted in 1.8% of lenalidomide maintenance treated TE patients. In addition, lenalidomide maintenance reduced the myeloma mortality rate, with 16.6% of patients dying because of progressive disease, compared to 22.6% of the TE patients being observed. Overall, the data in the TE patients is positive, with improved survival and a low risk of death due to secondary carcinogenesis. With increasing number of patients receiving lenalidomide maintenance routinely post ASCT, these data provide reassurance.

TNE patients have the highest SPM incidence, particularly those receiving lenalidomide maintenance, where the overall incidence was 15.4%, compared to 10% in those being observed (p = 0.10). The highest incidence was noted in the older TNE patients, defined as those >74 years, where the overall incidence was 22.5% in those receiving lenalidomide maintenance and 13.9% in those being observed (p = 0.09). In the general UK population, cancer incidence per 100 person years is noted to be between 0.66 and 2.02 for patients aged between 70 and 84, far lower than the overall rate of between 3.1 and 6.1 for the TNE patients, Table 1.^13^ Although not statistically significant, this is important and clinicians and patients should be aware of the high SPM incidence in older patients, so that signs and symptoms are investigated appropriately. The lack of statistical significance may relate to low numbers of TNE patients remaining on trial beyond 5 years (n = 86 lenalidomide maintenance, n = 68 observation) but irrespective of this, the data suggests that second carcinogenesis is inherently high in elderly patients with myeloma, whether maintenance strategies are used or not. A predisposition to carcinogenesis in older patients is also suggested by the shorter time to SPM development in the TNE pathway, compared to the TE patients (33.7 months TNE, 41.9 months TE). The reason is likely multifactorial, including altered immunity, cytotoxic treatment, and the accumulation of genetic aberrations during the patient life-course.

Haematological SPM incidence was low, with an overall incidence of 1.1% for all trial participants. Furthermore, the incidence remained low in lenalidomide maintenance patients, with an incidence of 2.8% in TE patients and 1.4% in TNE patients. The higher incidence rate in TE patients may be linked to the use of melphalan ASCT conditioning, consistent with previous meta-analysis.^8^ It was also noted that there was a greater incidence of hSPM in patients treated with lenalidomide at induction and maintenance, although this was mainly a feature in the TE patients where MDS incidence was 1.1%, again, possibly linked to melphalan. The increased incidence of solid tumours in TNE patients (5.5% vs 3% TE), but lower incidence of hSPM (0.8% vs 1.4% TE) suggest that melphalan is not contributing to solid tumour pathogenesis, as seems to be a contributory mechanism in hSPM development. The median time to hSPM development was 51.8 months in the TE patients and 42.3 months in the TNE patients. This was longer than the time for NMSC (42 months TE, 37.3 months TNE) and solid tumour diagnosis (38.8 months TE, 32 months TNE). The reason for this is not clear but indicates that the development of hSPM is complex. We have previously shown there is not a genetic signature associated with malignant plasma cell mutagenesis in patients who relapse post lenalidomide maintenance, but this has not been extensively explored as a possible cause for secondary carcinogenesis.^14^ In both the TE and TNE pathways, the use of lenalidomide maintenance did not result in a shorter time to SPM development.

Solid SPMs developed in TNE patients were consistent with the general population, with colon, prostate, breast, and lung frequently diagnosed, Fig. 2c. In TE patients, colon and breast were common, although MDS and AML were noted to affect more patients than the other solid tumour types, Fig. 2b. The impact of pre-existing risk factors for carcinogenesis in myeloma patients may be illustrated by the finding that 98/318 (31%) patients who developed an SPM, did so during the induction phase. It is unlikely that treatment related mutagenesis impacted SPM development so early in the disease course. As seen in hSPM, most solid tumour incidence was greater in patients treated with both lenalidomide at presentation and relapse. This could represent a cumulative effect of lenalidomide exposure as a risk factor for SPM development, possibly due to a direct impact on the genetics of non-myeloma cells or because of altered immune surveillance.

Almost a third of SPM patients developed a NMSC. This was particularly marked in the TNE patients, where 73 of the 180 (41%) SPM patients were diagnosed with a NMSC, compared to 33/138 (24%) TE patients. In both pathways, most cases were diagnosed in patients receiving lenalidomide maintenance. The data reveals the predisposition to these malignancies in immunocompromised patients.

The overall incidence of SPM was highest in patients who received lenalidomide at both induction and maintenance. This was however only significant in TNE patients treated at both time points, who had received fewer cycles than the TE group (median 34 cycles TE vs 27 TNE). This suggests that the risk of SPM is not conclusively linked to accumulative dose in all patient groups. The benefit of continuous lenalidomide in patients with long-term remissions is a consideration for future trial design.

It is important to note some potential confounding factors when interpreting these data. Patients being observed may be reviewed less frequently and as a result, there could be the potential for the under-reporting of SPMs. The trial data also indicates an overall survival benefit in lenalidomide maintenance treated TE patients and therefore this group of patients will have more time to develop an SPM, when compared to those being observed. The study is also open label and therefore patients receiving lenalidomide maintenance would have been counselled regarding the small SPM risk. This could result in increased vigilance and reporting of signs and symptoms of potential SPM in the lenalidomide maintenance patients. Of note, we also pooled the lenalidomide and lenalidomide + vorinostat data, due to low numbers of combination maintenance patients, poor tolerance of vorinostat in the combination maintenance setting and a lack of outcome improvement, meaning a separation of the analysis was not indicated.^15^ We did however show in Table 1, that the SPM incidence confidence intervals overlapped, providing reassurance that there was no additional SPM risk caused by the addition of vorinostat. The data for patients receiving combination carfilzomib and lenalidomide (KCRd) induction is not as mature as the data for patients who received lenalidomide (RCd) or thalidomide (CTd), but the 100 person-year SPM incidence was comparable, suggesting that the addition of carfilzomib is not associated with an increased SPM risk in this context. We acknowledge that we have not directly assessed the impact of cyclophosphamide, lenalidomide or bortezomib on SPM development in this manuscript. Bortezomib was used in a small cohort of poorly responding patients only, and therefore this would not be a representative group to assess for SPM risk.^16^ Cyclophosphamide was given to all patients, therefore a control variable in the trial, and has not been implicated as an overt SPM risk factor in meta-analysis.^8^^,^^10^ Thalidomide is poorly tolerated as a maintenance strategy and with newer, more effective agents available, long-term administration is unlikely to form a treatment strategy moving forward.^17^ Previous analyses have not shown a higher SPM incidence, particularly hSPM, in patients receiving thalidomide compared to lenalidomide.^18^

The data provide reassurance that lenalidomide maintenance is associated with a low incidence of hSPM, low risk of SPM death and improved survival, particularly post ASCT. There is an increased SPM incidence in lenalidomide maintenance patients, but a third of cases are low risk, NMSC. The increased SPM incidence is not statistically significant in TNE patients, illustrating the predisposition to carcinogenesis in older patients with myeloma, irrespective of whether lenalidomide maintenance is used or not. Patients treated with long-term lenalidomide need to be counselled regarding the signs and symptoms of possible SPM, particularly skin cancers. Clinic review should include a history and examination, focused on identifying possible SPM, so intervention can be made appropriately and in a timely fashion.

## Contributors

The Myeloma XI study design and protocol was developed by GJM, GHJ and FED. DAC, TM and JRJ analysed and interpreted the data presented in the manuscript. Statistical analysis was undertaken by DAC and TM. JRJ and DAC created the figures and tables. JRJ, GHJ, KDB and RGO reviewed SPM cases. JRJ drafted the first version of the manuscript. JRJ, DAC, TM, CP, FED, RS, AB, MWJ, MFK, CO, MR, MTD, RGO, KDB, GC, GJM and GHJ contributed to amendments and approval of the final version for submission.

## Data sharing statement

All requests for data not provided in the manuscript and supplementary material must be requested from the corresponding author in the first instance. Requests will be discussed with the trial management group and approval sought from the independent trial steering committee. Only requests that have a methodologically sound proposal will be considered. All shared data will be de-identified.

## Declaration of interests

DAC is a DSMB statistician for academically led investigator-initiated study in multiple myeloma; CP has received honoraria, undertaken consultancy and received research funding from BMS/Celgene; AB has received honoraria from BMS/Celgene, GSK, Sanofi, Amgen, Takeda and Janssen, received travel support from Amgen, Sanofi and BMS/Celgene, and undertaken consultancy for BMS/Celgene, Janssen, Takeda and Sanofi. MWJ has received honoraria from Pfizer, Janssen, BMS and Sanofi, and received meeting support from Janssen and Merinari Stemline; MFK has received research funding from BMS/Celgene and Janssen, honoraria from BMS/Celgene, Takeda and Abbvie and undertaken consultancy for Adaptive, Abbvie, BMS/Celgene, Pfizer, GSK, Karyopharm and Seattle Genetics; RGO has undertaken consultancy from Janssen and Beigene, received honoraria from Astra Zeneca, Janssen and Beigene, and received meeting support from Beigene; GHJ has received honoraria and research funding from BMS/Celgene. Celgene corporation, Merck Sharpe and Dohme and Amgen have provided unrestricted educational grants relating to this study, paid to the Clinical Trials Research Unit, University of Leeds.

## Appendix A Supplementary data

Myeloma XI Study Protocol
