# Supplementary material for: Maintenance lenalidomide in newly diagnosed transplant eligible and non-eligible myeloma patients; profiling second primary malignancies in 4358 patients treated in the Myeloma XI Trial
Source: eClinicalMedicine. 2023 Jul 27;62:102099. doi: 10.1016/j.eclinm.2023.102099 (PMC10404862; doi:10.1016/j.eclinm.2023.102099)
Supplement: Supplementary Figures S1 and S2 and Tables S1–S5 [file mmc2.docx]

**Supplementary Figures**

**Supplementary Figure 1: Myeloma XI Trial protocol**


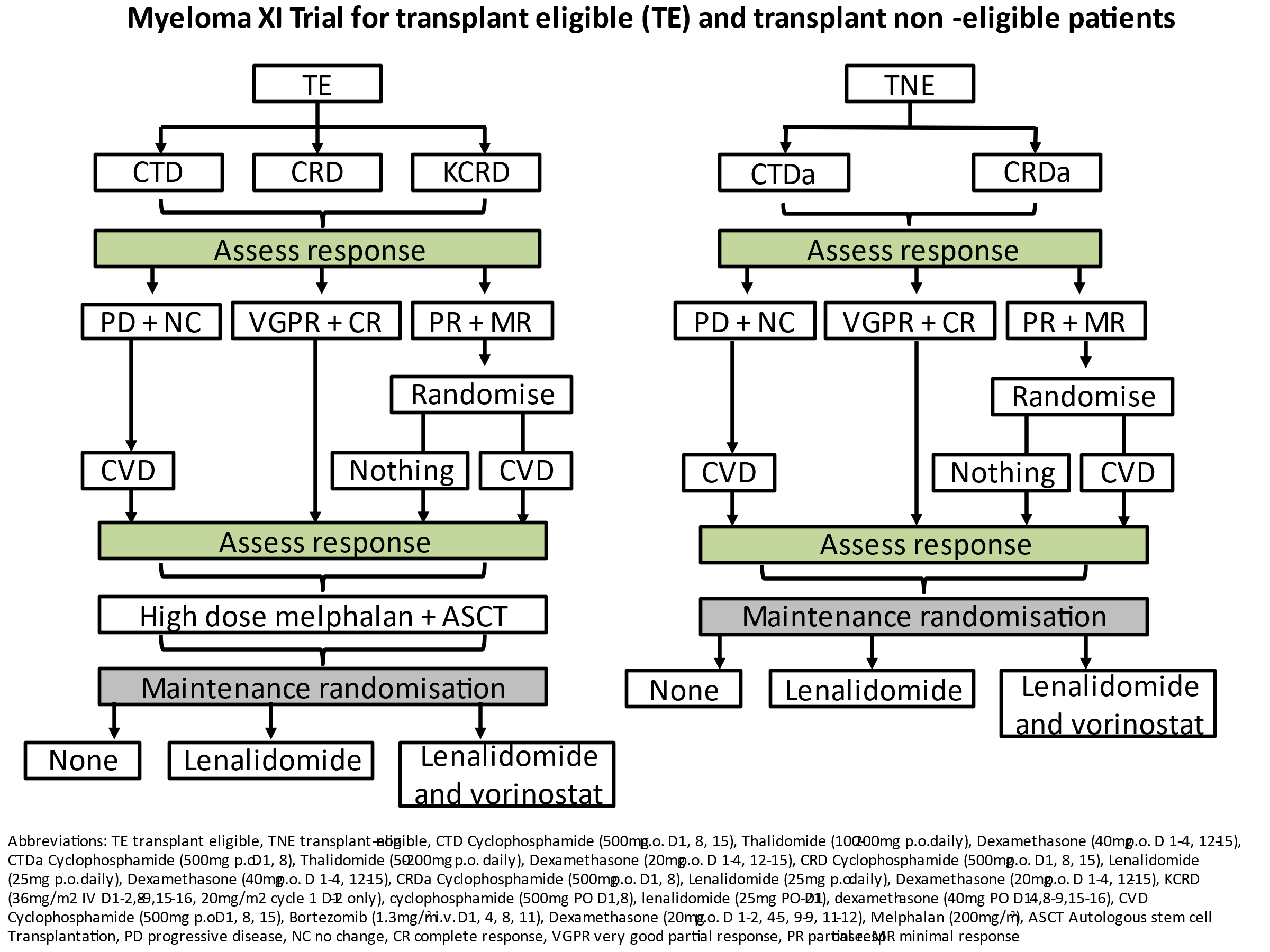


Myeloma XI Trial protocol showing the possible treatment randomisations for transplant eligible and non-eligible patients. Transplant eligible patients were randomised to thalidomide, lenalidomide or carfilzomib induction regimes, whilst non-eligible patients were randomised between thalidomide or lenalidomide containing regimes. Patients who achieved less than a very good partial remission may have received velcade based consolidation prior to ASCT (TE), or maintenance randomisation (TNE). Patients who achieved at least a minimal response were eligible for maintenance randomisation, which was between observation only, lenalidomide or lenalidomide + vorinostat.

**Supplementary Figure 2: SPM cumulative incidence according to age group in TNE patients**

**Supplementary Figure 2a: SPM incidence in TNE patients aged ≤74**


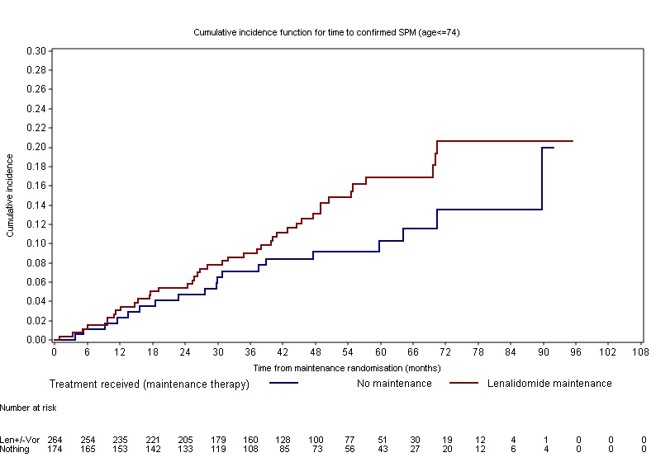


**Supplementary Figure 2b: SPM incidence in TNE patients aged >74**


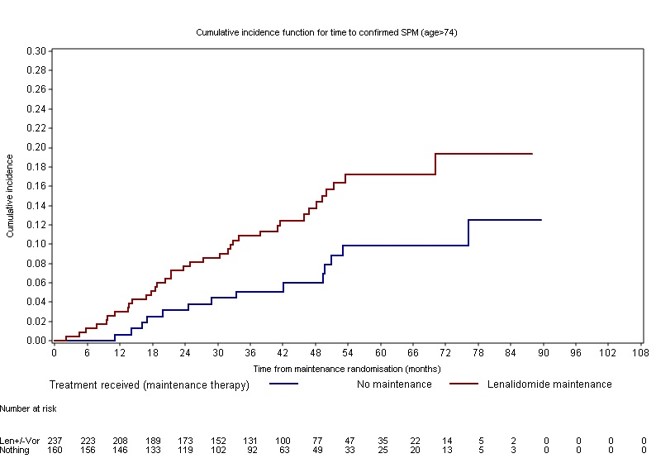


**Supplementary Figure 2a**. The cumulative incidence (CI) for TNE patients aged ≤74 according to maintenance randomisation. The SPM CI in patients ≤74 who were randomised to observation was 10.8% at 5 years, compared to 16.6% n those who received lenalidomide +/- vorinostat (Pepe Mori p=0.30).

**Supplementary Figure 2b.** The CI for TNE patients aged >74 according to maintenance randomisation. The SPM CI in patients >74 randomised to observation was 9.5% at 5 years, compared to 17.7% in those who received lenalidomide +/- vorinostat (Pepe Mori p=0.09).

**Supplementary Tables**

**Supplementary Table 1: Induction and maintenance allocation for all trial patients who received at least one dose of study drug**

| Treatment | Regime | Median age (range) | Patients (n) |
| --- | --- | --- | --- |
| Induction | CTD | 61 (29-74) | 1008 |
|  | CRD | 61 (28-75) | 1014 |
|  | KCRD | 60 (33-75) | 510 |
|  | CTDa | 74 (54-89) | 910 |
|  | CRDa | 74 (60-92) | 916 |
| Median follow-up since trial entry | | | 60 months (IQR 47-76) |
| Maintenance | Lenalidomide | 66 (29-89) | 1368 |
|  | Observation | 66 (30-90) | 906 |
| Median follow-up from maintenance randomisation | | | 52 months (IQR 38-69) |
| Abbreviations: CTD, cyclophosphamide, thalidomide and dexamethasone; CRD, lenalidomide, cyclophosphamide and dexamethasone; KCRD, carfilzomib, cyclophosphamide, lenalidomide and dexamethasone; IQR, interquartile range; vori, vorinostat. | | | |

Supplementary table 1: The number of patients treated according to induction and maintenance randomisation in both transplant eligible and non-eligible pathways. The table also shows the median age, including the range, for all patient groups. The median follow-up from maintenance randomisation was 52 months and 60 months from induction randomisation.

**Supplementary Table 2: Median follow-up according to treatment received.**

| **Pathway** | **Treatment** | **Median (IQR) - months** |
| --- | --- | --- |
| **TE** | **CTD** | **67 (49-83)** |
|  | **CRD** | **68 (49-82)** |
|  | **KCRD** | **45 (37-50)** |
|  | **Observation** | **50 (33-69)** |
|  | **Lenalidomide +/- vorinostat** | **45 (33-63)** |
|  |  |  |
| **TNE** | **CTDa** | **61 (48-78)** |
|  | **CRDa** | **61 (48-77)** |
|  | **Observation** | **61 (44-76)** |
|  | **Lenalidomide +/- vorinostat** | **54 (42-74)** |

Supplementary Table 2. The median follow-up for all regimes received according to transplant eligible and non-eligible groups. The median follow up and interquartile ranges were well matched for all comparisons, apart from for KCRd, which was added later in the trial.

Abbreviations: CTD, cyclophosphamide, thalidomide and dexamethasone; CRD, lenalidomide, cyclophosphamide and dexamethasone; KCRD, carfilzomib, cyclophosphamide, lenalidomide and dexamethasone; a, attenuated; IQR, interquartile range

**Supplementary table 3: Haematological malignancies diagnosed according to treatment received.**

**3a. Haematological SPM according to treatment in the TE Pathway**

|  | **CTD** | | | **CRD** | | | **KRCD** | | | **Cons** | |
| --- | --- | --- | --- | --- | --- | --- | --- | --- | --- | --- | --- |
|  | **Induction** | **Len** | **Obs** | **Induction** | **Len** | **Obs** | **Induction** | **Len** | **Obs** | **No VCD** | **VCD** |
| **AML*** | **2** | **1** |  | **1** | **4** |  |  |  |  |  |  |
| **B-ALL** |  | **2** |  |  |  |  |  |  |  |  |  |
| **CML** |  |  |  |  | **1** |  |  |  |  |  |  |
| **Burkitts** |  |  |  |  | **1** |  |  |  |  |  |  |
| **DLBCL** |  |  | **1** | **1** | **2** |  |  |  |  |  |  |
| **MDS*** | **1** | **5** | **1** | **2** | **5** |  |  | **1** |  | **3** |  |
| **Hodgkin lymphoma** |  |  |  |  | **1** |  |  |  |  |  |  |
| **T-ALL** |  | **1** |  |  |  |  |  |  |  |  |  |
| **ATCL** |  |  |  | **1** |  |  |  |  |  |  |  |

* One patient developed MDS, which progressed to AML at a later date.

**3b. Haematological SPM according to treatment in the TNE pathway**

|  | **CTDa** | | | **CRDa** | | | **Consolidation** | |
| --- | --- | --- | --- | --- | --- | --- | --- | --- |
| **SPM** | **Induction** | **Len** | **Obs** | **Induction** | **Len** | **Obs** | **No VCD** | **VCD** |
| **AML** |  | **1** | **1** | **1** | **1** | **1** |  |  |
| **B-ALL** |  |  |  |  | **2** |  |  |  |
| **MDS** | **1** | **1** |  |  | **1** |  | **1** |  |
| **DLBCL** |  |  |  | **2** |  |  |  |  |

Supplementary table 3a: Number and type of haematolgical SPM developed according to induction and maintenance therapy received in transplant eligible patients.

Supplementary table 3b: Number and type of haematolgical SPM developed according to induction and maintenance therapy received in transplant non-eligible patients.

Abbreviations: MDS, myelodysplastic syndrome; AML, acute myeloid leukaemia; B-ALL, B lymphocyte acute lymphoblastic leukaemia; DLBCL, diffuse large B cell lymphoma; CML, chronic myeloid leukaemia; T-ALL, T lymphocyte acute lymphoblastic leukaemia; Cons, consolidation phase; CTD, cyclophosphamide, thalidomide and dexamethasone; CRD, lenalidomide, cyclophosphamide and dexamethasone; KCRD, carfilzomib, cyclophosphamide, lenalidomide and dexamethasone; len, lenalidomide; obs, observation; a, attenuated; VCD, velcade, cyclophosphamide, dexamethasone,

**Supplementary table 4: Solid malignancies diagnosed according to pathway**

**Table 4a. Solid SPM according to treatment in the TE pathway**

|  | **CTD** | | | **CRD** | | | **KRCD** | | | **Cons** | |
| --- | --- | --- | --- | --- | --- | --- | --- | --- | --- | --- | --- |
| **SPM** | **Induction** | **Len** | **Obs** | **Induction** | **Len** | **Obs** | **Induction** | **Len** | **Obs** | **No VCD** | **VCD** |
| **Breast** |  | **1** | **2** | **3** | **1** | **1** | **1** |  |  |  |  |
| **Colon** | **1** | **1** |  | **1** | **1** |  |  |  |  | **1** |  |
| **Unknown primary** | **1** | **2** |  |  | **1** |  | **2** | **1** |  |  |  |
| **GIST** |  |  |  |  |  |  |  | **1** |  |  |  |
| **Liposarcoma** |  |  |  |  | **1** |  |  |  |  |  |  |
| **Liver** |  | **1** |  |  |  |  |  |  |  |  |  |
| **Lung** | **2** | **2** |  |  | **1** |  |  |  |  |  |  |
| **Maxillary SCC** |  |  |  |  | **1** |  |  |  |  |  |  |
| **Melanoma** |  |  |  |  | **1** | **1** | **1** | **1** |  | **2** |  |
| **Meningioma** |  |  |  |  | **1** |  |  |  |  |  |  |
| **Oesophageal** |  | **1** |  |  |  | **1** |  | **1** |  |  |  |
| **Pancreas** | **1** |  |  |  |  | **1** |  |  |  |  |  |
| **Prostate** |  | **1** | **3** | **3** | **1** | **2** |  | **2** | **2** | **2** |  |
| **Rectum** |  |  | **1** | **1** | **1** |  |  |  |  |  |  |
| **Thyroid** |  |  |  |  | **1** |  |  |  |  |  |  |
| **Glioblastoma** |  |  | **1** |  |  |  |  |  |  |  | **1** |
| **Ovary** |  |  | **1** | **1** |  |  |  |  |  |  |  |
| **Testis** |  |  |  |  |  | **1** |  |  |  |  |  |
| **Caecum** |  |  |  |  |  |  | **1** |  |  |  |  |
| **Bladder** | **1** |  |  |  |  |  |  |  |  |  |  |
| **Mesothelioma** | **1** |  |  |  |  |  |  |  |  | **1** |  |
| **Sarcoma** | **1** |  |  |  |  |  |  |  |  |  | **1** |
| **Renal cell** | **1** |  |  |  |  |  |  |  |  |  |  |
| **Endometrial** | **1** |  |  |  |  |  |  |  |  |  |  |
| **Tonsil** |  |  |  |  |  |  |  |  |  | **1** |  |

**Table 4b. Solid SPM according to treatment in the TNE pathway**

|  | **CTDa** | | | **CRDa** | | | **Consolidation** | |
| --- | --- | --- | --- | --- | --- | --- | --- | --- |
| **SPM** | **Induction** | **Len** | **Obs** | **Induction** | **Len** | **Obs** | **No VCD** | **VCD** |
| **Colon** | **5** | **5** | **3** | **1** | **1** |  |  |  |
| **Prostate** |  | **1** |  |  | **3** | **2** |  |  |
| **Breast** | **3** | **2** | **1** | **2** | **1** | **1** |  | **1** |
| **Lung** | **1** | **3** | **1** | **3** | **1** |  | **1** | **1** |
| **Oesophageal** | **1** | **2** |  |  | **2** | **1** |  |  |
| **Bladder** | **3** |  | **2** |  | **2** |  |  |  |
| **Unknown primary** |  |  | **1** |  | **2** | **1** | **1** |  |
| **Melanoma** | **2** | **1** |  | **2** | **3** | **1** |  |  |
| **Meningioma** | **1** |  |  |  | **1** |  |  |  |
| **Pancreas** | **2** |  |  | **1** | **1** | **1** |  |  |
| **Renal cell** | **1** |  | **2** | **2** |  |  |  |  |
| **Anal** |  |  |  |  | **1** |  |  |  |
| **Caecum** | **1** | **1** |  |  |  |  |  |  |
| **Endometrial** |  |  |  | **1** | **1** |  |  |  |
| **GIST** |  | **1** |  |  |  |  |  |  |
| **Glioblastoma** |  |  |  |  | **1** |  |  |  |
| **Liver** | **1** | **1** |  | **1** |  |  |  |  |
| **Mesothelioma** |  |  |  |  | **1** |  |  | **1** |
| **leukaemia (mixed)** |  |  |  |  | **1** |  |  |  |
| **Supraglotteal** |  |  |  |  | **1** |  |  |  |
| **Bile duct** |  |  | **1** |  |  |  |  |  |
| **Gastric** |  |  |  | **2** |  | **1** |  |  |
| **Ovary** | **1** |  |  | **1** |  | **1** |  |  |
| **Sarcoma** | **1** |  |  |  |  |  |  |  |
| **Rectum** | **1** |  |  | **1** |  |  |  |  |
| **Fibroxanthoma** |  |  |  |  |  |  |  | **1** |

Supplementary table 4a: Number and type of solid SPM developed according to induction and maintenance therapy received in transplant eligible patients.

Supplementary table 4b: Number and type of solid SPM developed according to induction and maintenance therapy received in transplant non-eligible patients.

Abbreviations: GIST, gastrointestinal stromal tumour; SCC, squamous cell carcinoma; BCC, basal cell carcinoma; CTD, cyclophosphamide, thalidomide and dexamethasone; CRD, lenalidomide, cyclophosphamide and dexamethasone; KCRD, carfilzomib, cyclophosphamide, lenalidomide and dexamethasone; len, lenalidomide; obs, observation; VCD, velcade, cyclophosphamide, dexamethasone; a, attenuated.

**Supplementary table 5: Non-melanoma skin cancers diagnosed according to pathway**

**Table 5a. Non-melanoma skin cancer SPM according to treatment in the TE pathway**

|  | **CTD** | | | **CRD** | | | **KRCD** | | | **Cons** | |
| --- | --- | --- | --- | --- | --- | --- | --- | --- | --- | --- | --- |
| **SPM** | **Induction** | **Len** | **Obs** | **Induction** | **Len** | **Obs** | **Induction** | **Len** | **Obs** | **No VCD** | **VCD** |
| **BCC** | **3** | **6** | **1** | **2** | **6** |  |  | **2** |  | **3** |  |
| **SCC** | **2** | **3** |  | **1** | **4** |  |  | **3** |  | **1** |  |

**Table 5b. Non-melanoma skin cancer SPM according to treatment in the TNE pathway**

|  | **CTDa** | | | **CRDa** | | | **Consolidation** | |
| --- | --- | --- | --- | --- | --- | --- | --- | --- |
| **SPM** | **Induction** | **Len** | **Obs** | **Induction** | **Len** | **Obs** | **No VCD** | **VCD** |
| **SCC** | **4** | **15** | **5** | **7** | **12** | **4** |  |  |
| **BCC** | **4** | **8** | **2** | **5** | **8** | **4** | **1** | **2** |

Supplementary table 5a: Number and type of solid SPM developed according to induction and maintenance therapy received in transplant eligible patients.

Supplementary table 5b: Number and type of solid SPM developed according to induction and maintenance therapy received in transplant non-eligible patients.

Abbreviations: SCC, squamous cell carcinoma; BCC, basal cell carcinoma; CTD, cyclophosphamide, thalidomide and dexamethasone; CRD, lenalidomide, cyclophosphamide and dexamethasone; KCRD, carfilzomib, cyclophosphamide, lenalidomide and dexamethasone; len, lenalidomide; obs, observation; VCD, velcade, cyclophosphamide, dexamethasone; a, attenuated.
